# Case report of successful conservative management of neonatal gastrointestinal perforation and intestinal stenosis

**DOI:** 10.1097/MD.0000000000050502

**Published:** 2026-09-18

**Authors:** Xiaoqing Li, Enfu Tao, Huafei Huang

**Affiliations:** a Department of Neonatology, Jiaxing Maternity and Child Health Care Hospital, Jiaxing, Zhejiang Province, China; b Department of Neonatology and NICU, Wenling Maternal and Child Health Care Hospital, Wenling, Zhejiang Province, China.

**Keywords:** case report, conservative treatment, intestinal stenosis, necrotizing enterocolitis, neonatal gastrointestinal perforation, pneumoperitoneum, sepsis

## Abstract

**Rationale:**

Neonatal gastrointestinal perforation (NGP) is a life-threatening condition requiring urgent intervention. While exploratory laparotomy remains the standard treatment, conservative management offers an alternative when surgery is not feasible. This case demonstrates the successful use of comprehensive conservative therapy in a high-risk infant with NGP and suspected intestinal stenosis.

**Patient concerns:**

A 6-day-old, small-for-gestational-age male infant presented with vomiting, abdominal distension, and respiratory distress. Clinical deterioration led to the diagnosis of pneumoperitoneum, sepsis, and suspected necrotizing enterocolitis.

**Diagnoses:**

NGP secondary to suspected necrotizing enterocolitis, with complications including septic shock, purulent meningitis, scrotal abscess, zinc deficiency, and malnutrition.

**Interventions:**

Following parental refusal of laparotomy, conservative management was initiated, including peritoneal drainage, gastrointestinal decompression, broad-spectrum antibiotics, inotropic support, and parenteral nutrition. Nutritional support included zinc supplementation and gradual enteral feeding.

**Outcomes:**

The infant survived after 134 days of hospitalization, despite multiple complications. Long-term follow-up at 8 months revealed neurodevelopmental delays (developmental quotient 61), though physical growth was adequate with successful transition to complementary feeding.

**Lessons:**

This case confirms that conservative management with peritoneal drainage and intensive multidisciplinary support can be a viable option for NGP, even in complex scenarios where surgery is not possible. It underscores the importance of vigilant monitoring, nutritional management, and awareness of potential long-term neurodevelopmental sequelae in such cases.

## 1. Introduction

Neonatal gastrointestinal perforation (NGP) is a severe, life-threatening condition requiring immediate diagnosis and treatment.^[1]^ It presents suddenly, progresses quickly, and has a high mortality rate.^[2,3]^ NGP arises from 2 main causes: inflammatory/ischemic types like necrotizing enterocolitis (NEC) and spontaneous intestinal perforation (SIP), which have similar symptoms and diagnostic challenges, and obstructive types like malrotation with volvulus and meconium obstruction, which often require surgery.^[2,4–8]^ Complications such as sepsis and secondary intestinal obstruction worsen the situation, complicating management and leading to poor outcomes.^[2,9]^

Traditionally, exploratory laparotomy has been the gold standard for treating NGP by identifying and managing perforation sites.^[10,11]^ Recent studies, however, suggest peritoneal drainage (PD) as a viable alternative, showing no significant differences in complications, recovery time, hospital stay, neurodevelopmental outcomes, or mortality compared to laparotomy.^[12,13]^ Additionally, conservative management includes gastrointestinal decompression, parenteral nutrition, and broad-spectrum antibiotics to stabilize the patient and promote spontaneous sealing of the perforation.^[11]^

This case report discusses a neonate with NGP likely caused by NEC, complicated by secondary sepsis, septic shock, and intestinal obstruction. The condition was successfully managed with PD and conservative treatment after the family opted against surgery following a detailed discussion of risks and outcomes. The approach led to positive results, effectively addressing gastrointestinal perforation and its complications, demonstrating the efficacy of these methods in similar cases.

## 2. Case presentation

**Figure 1.**
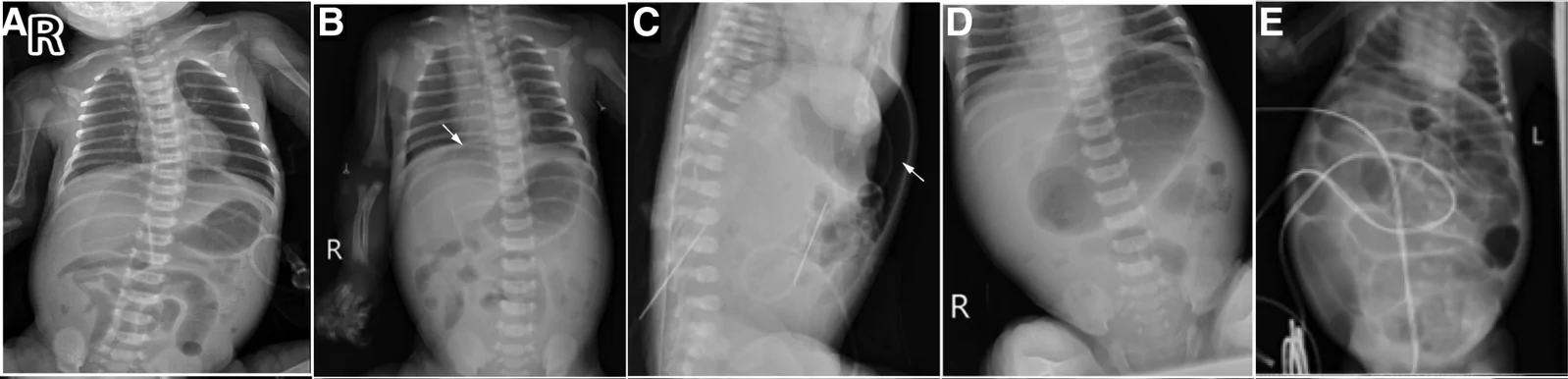
Representative abdominal radiographic findings in a term-born small-for-gestational-age infant with neonatal gastrointestinal perforation. (A) Pneumatosis intestinalis and segmental gas accumulation in the intestinal tract; (B) anteroposterior view of pneumoperitoneum; (C) lateral view of pneumoperitoneum; (D) anteroposterior view; and (E) intestinal dilation.

A 6-day-old male neonate was transferred to our hospital after experiencing vomiting and abdominal distension for half a day. He had initially been admitted to another hospital for “transient tachypnea of the newborn and neonatal hyperbilirubinemia” due to shortness of breath and a peripheral capillary oxygen saturation of 77%. Just before discharge, he began biliary vomiting and developed abdominal distension, with a heart rate of 180 beats per minute. An emergency complete blood count showed a white blood cell (WBC) count of 4.7 × 10^9^/L and a high-sensitivity C-reactive protein level of 16.83 mg/L. An abdominal X-ray indicated pneumatosis intestinalis and segmental gas accumulation, but no portal venous gas was found (Fig. 1A). After receiving treatment at a local hospital, including antibiotics and gastrointestinal decompression, the patient’s vomiting and abdominal distension persisted, leading to their transfer to our facility. The patient, a firstborn from a primigravida, was delivered via cesarean section at 39 weeks and 5 days, weighing 2570g. Both amniotic fluid and placenta were normal, and Apgar scores at 1, 5, and 10 minutes were all 10. The family history revealed social issues, with the mother being an 18-year-old unmarried woman and the father a 21-year-old unemployed man, amidst noted family disputes. There was no history of infectious or genetic diseases.

**Figure 2.**
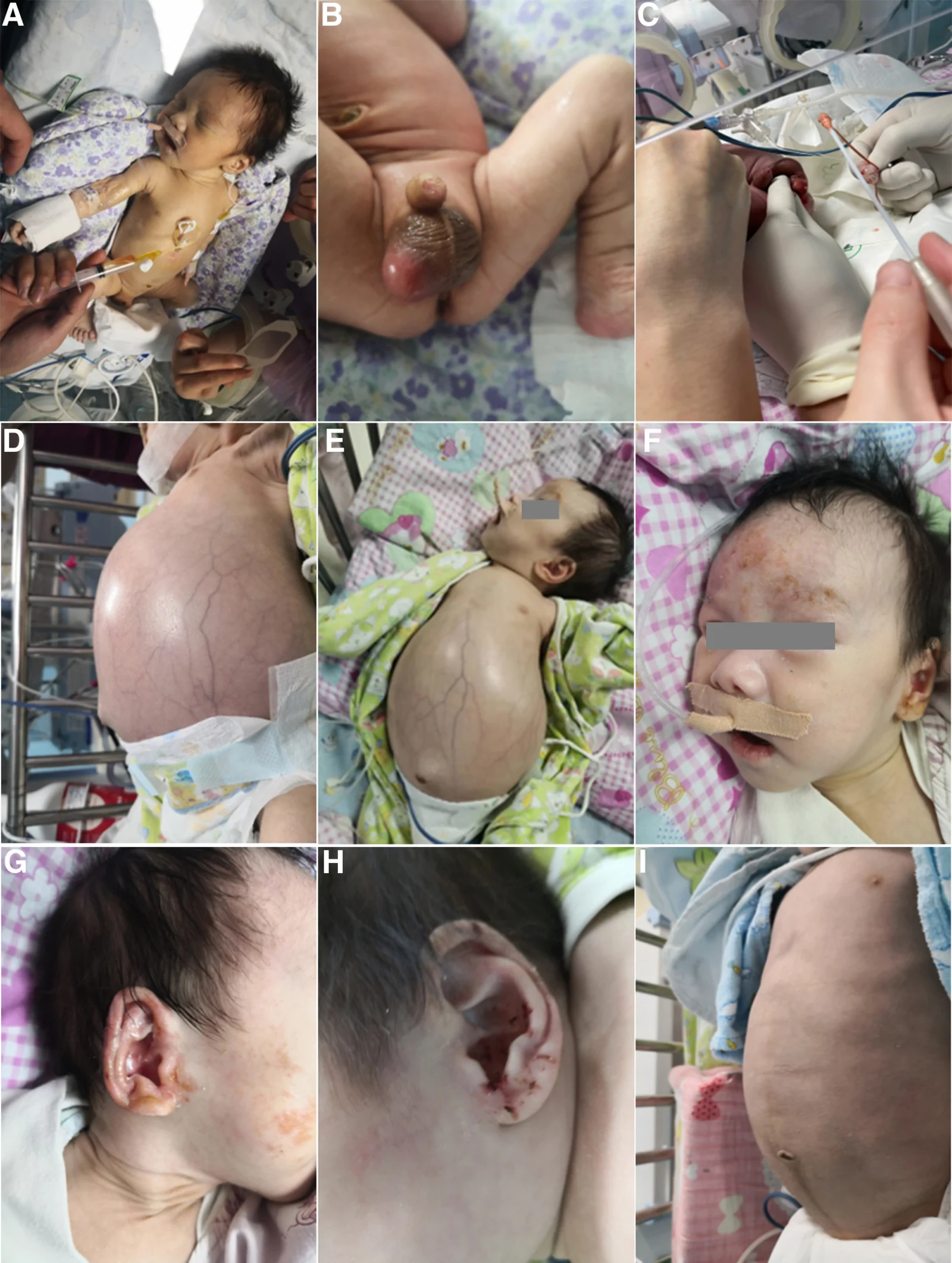
Image of clinical features of the term-born small-for-gestational-age infant with neonatal gastrointestinal perforation. (A) Abdominal paracentesis. Abdominal paracentesis drained 15 mL of gas and 2 mL of yellowish serous fluid at day 1. (B) Scrotal abscess. The patient developed a right scrotal abscess at day 14. (C) Scrotal puncture. 2 mL of purulent secretion was slowly extracted, and it cultured *Enterobacter cloacae*. (D) Severe abdominal distension with prominent abdominal wall veins at day 43. (E) Severe abdominal distension at day 48. (F–H) Erythematous, crusted, and scaled lesions on the forehead, face, the right ear, and the left ear at day 49, respectively. (I) Abdominal appearance at day 110.

Upon admission, the patient exhibited a temperature of 38.6°C, blood pressure of 69/51 mm Hg, respiratory rate of 50 breaths per minute, heart rate of 180 beats per minute, poor responsiveness, opisthotonos, pallor, a flat anterior fontanel, clear breath sounds, rhythmic heart sounds, significant abdominal distension, absent bowel sounds, crying upon palpation, cool extremities, and a capillary refill time of 6 seconds, with peripheral capillary oxygen saturation at 95%. These findings indicated an acute surgical abdomen. Emergency chest and abdominal X-rays revealed pneumoperitoneum (Fig. 1B and C). Abdominal ultrasound showed ascites but no mesenteric vascular issues. Blood tests indicated no significant abnormalities, with a complete blood count revealing a WBC count of 4.3 × 10^9^/L, neutrophils at 67.8%, lymphocytes at 19.7%, and high-sensitivity C-reactive protein at 34.1 mg/L. Due to suspected gastrointestinal perforation, sepsis, and septic shock, immediate treatment included saline for volume expansion, dopamine for circulation, and empirical antibiotics (meropenem and vancomycin). Gastrointestinal decompression, nil per os (NPO) status, and parenteral nutrition were also initiated, and an exploratory laparotomy was deemed urgent. However, the family declined surgery, citing previous hospital errors as the cause of the pneumoperitoneum, and chose conservative management despite being warned of severe risks, including neonatal death. PD was performed at the bedside. The infant was placed in the supine position. After routine disinfection with iodophor, sterile gloves and drapes were applied. An 18-gauge intravenous cannula was inserted perpendicularly approximately 1 cm below the xiphoid process. A distinct loss of resistance was felt upon entering the peritoneal cavity, followed by connection to a 5 mL syringe, through which 15 mL of gas and 2 mL of yellowish serous fluid were slowly aspirated (Fig. 2A). The patient remained hemodynamically stable throughout the procedure. The cannula was then removed, and the puncture site was covered with sterile gauze. By the second day of admission, the pneumoperitoneum had resolved (Fig. 1D). A multidisciplinary team identified NEC as the likely cause of NGP. Table 1 outlines the diagnostic process. Biochemistry showed an albumin level of 21.8 g/L, indicating neonatal hypoproteinemia, prompting intravenous albumin supplementation. The cerebrospinal fluid (CSF) analysis revealed clear fluid, a weakly positive Pandy test, and 74.0 × 10^6^/L nucleated cells. CSF biochemistry showed aspartate aminotransferase (AST) at 13 U/L, lactate dehydrogenase at 46 U/L, chloride at 119.8 mmol/L, glucose at 2.06 mmol/L, and protein at 80.8 mg/dL. Despite the negative CSF culture, purulent meningitis was still considered. After effective anti-infective treatment, both the CSF routine and biochemistry returned to normal (Table S1, Supplemental Digital Content 1, and Table S2, Supplemental Digital Content 2). Subsequent diagnostic tests, including blood culture, liver and kidney function tests, *Ureaplasma urealyticum* ribonucleic acid, chlamydia trachomatis ribonucleic acid (nasopharyngeal aspirate), urine cytomegalovirus deoxyribonucleic acid, and thyroid function, all returned negative results. Echocardiography indicated an atrial septal defect with a diameter of 1.4 mm. Cranial ultrasound revealed no abnormalities.

**Table 1:** Diagnosis and differential diagnosis of gastrointestinal perforation in the patient.

Diagnosis	Key Considerations	Supporting evidence	Not supporting
NEC	Common clinical manifestation: abdominal distension, gastric retention, feeding intolerance, vomiting, and occasionally bloody stools. Development of overwhelming sepsis, septic shock, MODS, DIC, and death. Pneumatosis intestinalis and portal vein gas.	Biliary vomiting, abdominal distension, and mucus-bloody stools. Pneumatosis intestinalis on abdominal radiography. Risk factors: young mother, prenatal stress, low birth weight, perinatal asphyxia, ASD.	–
SIP	Characterized by an isolated intestinal perforation with relatively normal surrounding tissue, typically occurring at the antimesenteric border of the terminal ileum during the first week of life.	The timing and early clinical presentation.	Often in preterm infants, particularly those with extremely low birth weight, with seldom reported cases in full-term infants.
Hirschsprung disease	A rare congenital intestinal disorder characterized by the absence of ganglion cells in the myenteric and submucosal plexuses of the intestine. Delayed passage of the first meconium beyond 24 h. Common clinical manifestation: abdominal distension and vomiting.	Biliary vomiting, abdominal distension.	The patient passed the first meconium within 24 h.
Intestinal malrotation	A congenital anomaly characterized by the abnormal positioning of the intestines. Most common clinical manifestation: bilious vomiting, followed by abdominal distension. Ultrasound: abnormal position of the duodenum and superior mesenteric vein, with the characteristic “whirlpool sign.”	Bilious vomiting and abdominal distension.	Ultrasound revealed no abnormalities.
Meconium ileus and meconium plug syndrome	Delayed passage of meconium, causing bowel obstruction and leading to increased intraluminal pressure and potential perforation.	Bilious vomiting and abdominal distension.	No history of delayed meconium passage.

ASD = atrial septal defect, DIC = disseminated intravascular coagulation, MODS = multiple organ dysfunction syndrome, NEC = necrotizing enterocolitis, SIP = spontaneous intestinal perforation.

The patient remained on NPO and gastrointestinal decompression for 20 days, during which daily gastric drainage reached up to 36 mL of greenish fluid. On the 14th day of admission, the patient developed a right scrotal abscess (Fig. 2B), confirmed by ultrasound. Treatment involved aspiration of purulent fluid (Fig. 2C), which cultured *Enterobacter cloacae*. By day 20, abdominal distension had resolved, vomiting had ceased, bowel sounds were normal, and stool occult blood tests were negative. The patient tolerated glucose water feeding for 2 days without gastrointestinal symptoms. However, after 22 days of minimal enteral feeding with term formula, abdominal distension with intestinal loops, vomiting, and abdominal wall varicosities recurred, and X-rays indicated intestinal dilation (Figs. 1E and 2D), suggesting intestinal obstruction and suspected intestinal stenosis. No contrast enema was performed as the family declined. Conservative management included nasogastric decompression, strict NPO with total parenteral nutrition, and serial abdominal imaging. On day 48, the patient again developed severe abdominal distension (Fig. 2E). The patient was placed on NPO for another 62 days. On day 88, abdominal distension had resolved, vomiting had ceased, and bowel sounds were normal. Glucose water feeding was initiated for 5 days, and on day 93, minimal enteral feeding with term formula was restarted and slowly advanced, reaching full enteral nutrition by day 115.

By day 49, erythematous, crusted, and scaled lesions had appeared on the forehead, face, right ear, and left ear (Fig. 2F–H). Trace element analysis revealed a zinc level of 24.70 μmol/L (reference range = 46.27–128.39 μmol/L), indicating zinc deficiency. The patient was given oral zinc gluconate granules for supplementation. Follow-up zinc levels were 34.27 μmol/L on the 108th day of admission and 38.61 μmol/L on the 134th day of admission.

By day 110, the patient’s abdominal distension had significantly improved, with a flat and soft abdomen (Fig. 2I). On day 134 of hospitalization, the patient weighed 4770 g, which was below the 3rd percentile of the growth standards for boys under 7 years old in China,^[14]^ suggesting malnutrition. Throughout hospitalization, the patient experienced 2 episodes of nosocomial infections, which were treated with meropenem and amoxicillin–clavulanate, respectively. WBC counts ranged from 4.3 × 10^9^/L to 11.3 × 10^9^/L, with CRP peaking at 151.9 mg/L and platelet counts as low as 100 × 10^9/^L (Table 2). Blood gas analysis during hospitalization showed no significant abnormalities (Table S3, Supplemental Digital Content 3). Biochemistry during hospitalization was shown in Table S4, Supplemental Digital Content 4.

**Table 2:** Complete blood count results of the patient’s hospitalization.

Days after admission	WBC (×10^9^/L)	N%	L%	HGB (g/L)	PLT (×10^9^/L)	hs-CRP (mg/L)
1	4.3	67.8	19.7	157	119	34.1
1*	19.1	73.6	13.8	190	138	40.9
2	11.3	70.8	22.8	135	100	151.9
3	11.6	52.6	38.6	143	104	112.7
5	9.0	45.6	32.9	132	111	46.5
7	13.7	50.2	29.7	142	239	29.6
9	14.7	49.8	35.9	124	280	29.6
12	7.8	41.8	45.6	86	196	18.1
15	22.1	36.5	49.0	112	366	35.1
18	21.2	44.1	33.0	105	360	6.6
21	11.4	28.6	51.6	88	276	2.8
26	9.4	21.7	55.1	91	226	2.4
30	8.3	18.9	60.0	88	325	1.0
32	9.5	19.2	62.5	91	370	0.9
34	8.0	23.7	58.8	99	390	1.2
35	8.9	15.3	66.0	92	281	0.9
38	6.9	36.6	47.4	91	354	3.0
41	5.7	22.6	56.4	88	377	9.1
42	7.7	18.6	63.3	102	508	4.2
50	5.3	44.8	45.1	90	417	3.8
54	7.8	29.1	56.6	99	432	0.8
61	7.0	23.3	59.1	101	427	1.2
68	5.6	21.7	61.6	90	422	2.6
75	8.2	21.3	57.2	108	448	1.3
80	8.5	22.9	63.7	104	512	0.5
86	8.0	22.4	58.9	114	394	0.8
92	8.1	57.8	26.3	86	302	10.7
93	5.4	22.2	53.7	81	288	38.0
94	5.1	13.0	63.3	86	279	15.2
108	5.3	19.7	60.7	81	355	1.9
112	6.5	23.7	58.3	90	346	3.3
115	7.5	25.3	55.8	93	360	4.7
117	6.4	24.0	54.3	89	328	8.3
117*	8.1	17.3	60.1	101	397	24.9
134	6.7	31.9	52.7	94	304	3.7

HGB = hemoglobin, hs-CRP = high-sensitivity C-reactive protein, L% = lymphocyte percentage, N% = neutrophil percentage, PLT = platelet count, RBC = red blood cell count, WBC = white blood cell count.

* Re-examination in the afternoon.

The patient was ultimately diagnosed with gastrointestinal perforation (most likely secondary to NEC), peritonitis, intestinal obstruction, sepsis, septic shock, purulent meningitis, scrotal abscess (right side, *E cloacae* infection), small-for-gestational-age (SGA) infant, atrial septal defect, anemia, hypoproteinemia, zinc deficiency, and malnutrition. Through comprehensive treatment, including PD, volume expansion, dopamine to improve circulation, antibiotics, fluid replacement, parenteral nutrition, albumin support, vitamin A, D, and mineral supplementation, and gastrointestinal decompression, the infant’s condition ultimately improved. The patient was successfully discharged after 134 days of hospitalization. Follow-up at 8 months showed normal physical growth and development, with the addition of complementary foods and normal stools. Developmental quotient (DQ) was 61, indicating neurodevelopmental delays. The patient could walk independently at 1 year but was lost to follow-up thereafter.

## 3. Discussion

NGP is a life-threatening condition that requires prompt and effective management. This case underscores key aspects of NGP treatment, especially nonsurgical options due to the family’s refusal of surgery. The patient faced multiple complications, including peritonitis, bowel obstruction, sepsis, septic shock, neonatal purulent meningitis, scrotal abscess, zinc deficiency, and malnutrition, resulting in a lengthy hospital stay and negative neurodevelopmental effects. This rare case presents numerous clinical challenges that require further exploration.

Determining the etiology of NGP in this case was challenging due to the family’s refusal of exploratory laparotomy, which precluded histologic confirmation. NEC is the most likely cause based on several clinical features. The infant presented at 6 days of age with bilious vomiting, abdominal distension, and mucus-bloody stools – consistent with NEC in term infants, which typically occurs within the first days of life.^[15]^ Radiographically, the progression from pneumatosis intestinalis to pneumoperitoneum (Fig. 1A–C) paralleled the clinical deterioration to sepsis and septic shock. While SIP can also present with pneumoperitoneum in this age group, it is more common in preterm infants and does not typically cause extensive bowel necrosis or adverse long-term neurodevelopmental outcomes.^[16,17]^ The patient’s developmental delay at 8 months (DQ 61) aligns with the known association between NEC and adverse neurological outcomes, whereas SIP has not been linked to such risks.^[17]^ Thus, while definitive diagnosis awaits pathologic confirmation, the cumulative evidence strongly favors NEC as the underlying cause.

The successful outcome in this case supports PD as a viable alternative when laparotomy is not feasible. Systematic reviews and meta-analyses have found no significant differences in mortality between PD and laparotomy as initial surgical interventions for perforated NEC or SIP in preterm infants.^[18,19]^ However, evidence specific to term SGA infants is scarce. Our case suggests that this evidence may be extendable to a term SGA infant with NGP complicated by septic shock and meningitis, and implies that PD with intensive medical support may contribute to survival even in complex scenarios. Notably, the patient required a prolonged 134-day hospitalization, reflecting the significant morbidity that accompanies such conservative approaches. The key message is not that PD is superior to laparotomy, but rather that when surgery is refused or high-risk, PD combined with meticulous multidisciplinary care can serve as a life-saving alternative.

A noteworthy complication was the development of a right-sided scrotal abscess on day 14, from which *E cloacae* was cultured. Scrotal abscess in neonates is uncommon and typically occurs following abdominal surgery, umbilical infection, or sepsis.^[20–22]^ To the best of our knowledge, this is likely the first reported case of scrotal abscess caused by *E cloacae* secondary to NGP. The pathogenesis likely involves hematogenous spread from intraperitoneal infection or direct seeding via the patent processus vaginalis.^[23]^ High-resolution ultrasound effectively ruled out testicular torsion, which is the primary differential diagnosis.^[24]^ Although surgical incision and drainage is traditionally recommended, our case demonstrates that percutaneous aspiration with targeted antibiotics can be effective when surgery is declined, with ultrasound monitoring ensuring resolution.

The patient also developed recurrent abdominal distension and vomiting suggestive of intestinal obstruction, likely due to post-NEC stricture or adhesions. Intestinal strictures occur in approximately 19% of infants surviving acute NEC, typically presenting 2 to 7 weeks after the acute episode.^[25,26]^ However, not all post-NEC strictures require surgery; Tonkin et al^[27]^ reported spontaneous resolution in 28.6% of cases, with histopathology showing minimal fibrosis, suggesting that some strictures represent reversible inflammatory narrowing rather than fixed fibrotic obstructions. In our patient, conservative management – including gastrointestinal decompression, bowel rest, and gradual reintroduction of enteral feeds – successfully resolved the obstructive symptoms without surgical intervention. This outcome supports a trial of conservative management in hemodynamically stable infants with suspected post-NEC stricture.

Zinc deficiency was documented on day 49 (24.70 μmol/L; reference: 46.27–128.39 μmol/L), presenting as erythematous, crusted, scaling lesions on the face and ears that rapidly improved after supplementation. SGA infants are inherently vulnerable to zinc deficiency due to low birth stores, limited intake, and high growth demands.^[28]^ In this case, multiple factors converged: SGA status, intestinal perforation with impaired absorption, suspected intestinal stenosis, prolonged fasting, and extended total parenteral nutrition – all of which compounded the risk.^[28,29]^ The rapid resolution of skin lesions following zinc supplementation confirmed the diagnosis and highlights the importance of routine trace element monitoring in SGA infants with NGP requiring long-term bowel rest.

The neurodevelopmental delay observed at 8 months (DQ 61) likely reflects a “multiple-hit” model. SGA status alone is associated with lower cognitive outcomes compared with appropriate for gestational age infants.^[30,31]^ NEC adds another layer of risk, with systematic reviews demonstrating a significant association between NEC and neurodevelopmental impairment, particularly in surgically treated cases.^[32,33]^ In our patient, the inflammatory cascade – sepsis, septic shock, meningitis, and recurrent infections – likely amplified brain injury through systemic cytokine release that disrupts the blood-brain barrier and gut–brain axis.^[34,35]^ Prolonged antibiotic exposure and extended parenteral nutrition further compound these risks.^[34]^ The combination of these factors places this population at exceptionally high risk for adverse neurological outcomes. Early combined rehabilitation interventions, which have shown benefit in preterm infants,^[36]^ should be considered for SGA infants surviving NGP, although further research is needed.

Notably, several limitations should be acknowledged. First, the diagnosis of NEC remains presumptive due to the lack of histologic confirmation from exploratory laparotomy, which was declined by the family. Second, the suspected intestinal stenosis could not be radiologically confirmed as contrast imaging was refused. These diagnostic constraints may affect the interpretation of our findings, yet they importantly reflect the clinical challenges when parental decisions limit conventional diagnostic approaches in neonatal care.

## 4. Conclusion

This case report demonstrates that nonsurgical management, including PD and conservative care, can effectively treat severe NEC-induced NGP, offering a viable alternative to surgery in high-risk cases. Our findings indicate that with careful monitoring and supportive care, positive outcomes are possible even when surgery is not an option due to family preferences or high risks. Further research is needed to refine these methods for broader clinical use. Additionally, vigilance is necessary for complications in SGA infants post-NEC-induced NGP, such as scrotal abscess, intestinal stenosis, zinc deficiency, and malnutrition.

## Author contributions

**Data curation:** Xiaoqing Li, Enfu Tao, Huafei Huang.

**Formal analysis:** Xiaoqing Li, Enfu Tao, Huafei Huang.

**Investigation:** Xiaoqing Li.

**Resources:** Xiaoqing Li, Enfu Tao, Huafei Huang.

**Software:** Xiaoqing Li.

**Validation:** Xiaoqing Li, Enfu Tao, Huafei Huang.

**Conceptualization:** Enfu Tao.

**Funding acquisition:** Enfu Tao.

**Visualization:** Enfu Tao, Huafei Huang.

**Methodology:** Huafei Huang.

**Supervision:** Huafei Huang.

**Writing – original draft:** Xiaoqing Li, Enfu Tao, Huafei Huang.

**Writing – review & editing:** Xiaoqing Li, Enfu Tao, Huafei Huang.








