# Supplementary material for: Case report of successful conservative management of neonatal gastrointestinal perforation and intestinal stenosis
Source: Medicine (Baltimore). 2026 Sep 18;105(38):e50502. doi: 10.1097/MD.0000000000050502 (PMC13592972; doi:10.1097/MD.0000000000050502)
Supplement: Supplementary file 1 [file medi-105-e50502-s001.docx]

| Supplementary table 1 Cerebrospinal fluid routine during the patient's hospitalization | | | | | |
| --- | --- | --- | --- | --- | --- |
| Days after admission | Pandy's test | Nucleated cells  (× 10^6^/L) | Neutrophils (%) | Lymphocytes (%) | Monocytes (%) |
| 8 | weakly positive | 74 | 20 | 70 | 10 |
| 15 | weakly positive | 20 | - | - | - |
| 23 | weakly positive | 9 | - | - | - |
| 61 | negative | 1 | - | - | - |

The reference ranges for the measured values are as follows: Pandy protein qualitative test is negative, and nucleated cells are 0-30 × 10^6^/L. All cerebrospinal fluid samples were colorless and transparent.
