# Supplementary material for: Case report of successful conservative management of neonatal gastrointestinal perforation and intestinal stenosis
Source: Medicine (Baltimore). 2026 Sep 18;105(38):e50502. doi: 10.1097/MD.0000000000050502 (PMC13592972; doi:10.1097/MD.0000000000050502)
Supplement: Supplementary file 2 [file medi-105-e50502-s002.docx]

| Supplementary table 2 Cerebrospinal fluid biochemistry results during the patient's hospitalization | | | | | |
| --- | --- | --- | --- | --- | --- |
| Days after admission | AST (U/L), | LDH (U/L) | Glucose (mmol/L) | Chloride (mmol/L) | Protein (mg/dL) |
| 8 | 13 | 46 | 2.06 | 119.8 | 80.8 |
| 15 | 20 | 72 | 1.89 | 125 | 86.1 |
| 23 | 25 | 71 | 1.71 | 123.3 | 75.2 |
| 61 | 10 | 23 | 2.7 | 121.7 | 27.6 |

The reference ranges for the measured values are as follows: AST 0-20 U/L, LDH 0-40 U/L, glucose 1.12-2.24 mmol/L, chloride 111.0-123.0 mmol/L, and protein 20.0-120 mg/dL. AST: aspartate aminotransferase, LDH: lactate dehydrogenase.
