# Supplementary material for: Case report of successful conservative management of neonatal gastrointestinal perforation and intestinal stenosis
Source: Medicine (Baltimore). 2026 Sep 18;105(38):e50502. doi: 10.1097/MD.0000000000050502 (PMC13592972; doi:10.1097/MD.0000000000050502)
Supplement: Supplementary file 3 [file medi-105-e50502-s003.docx]

| Supplementary table 3 Blood gas analysis results during the patient's hospitalization | | | | | | | | | | | |
| --- | --- | --- | --- | --- | --- | --- | --- | --- | --- | --- | --- |
| Days after admission | pH | PCO2 (mmHg) | PO2 (mmHg) | HCO3^-^ (mmol/L) | Base excess (mmol/L) | Lactate (mmol/L) | Glucose (mmol/L) | Potassium (mmol/L) | Sodium (mmol/L) | Chloride (mmol/L) | Ionized calcium (mmol/L) |
| 1 | 7.45 | 30 | 91 | 20.8 | -1.8 | 2 | 6.4 | 4.5 | 136 | 114 | 1.2 |
| 1 | 7.45 | 33 | 76 | 22.6 | -0.3 | 2 | 3.9 | 4.6 | 136 | 110 | 1.07 |
| 2 | 7.41 | 33 | 99 | 20.6 | -2.7 | 1.4 | 2.6 | 3.9 | 137 | 112 | 1.31 |
| 3 | 7.43 | 32 | 85 | 20.9 | -2 | 3.2 | 2.7 | 4.5 | 135 | 108 | 1.33 |
| 5 | 7.42 | 40 | 92 | 25.7 | 1.7 | 0.8 | 4.3 | 3.9 | 137 | 107 | 1.33 |
| 9 | 7.47 | 32 | 122 | 23 | 0.4 | 1.6 | 4.9 | 4.7 | 137 | 110 | 1.26 |
| 12 | 7.44 | 36 | 104 | 23.9 | 0.5 | 0.6 | 4.9 | 4.3 | 138 | 111 | 1.34 |
| 21 | 7.48 | 30 | 90 | 21.7 | -0.9 | 1.4 | 4.2 | 4.3 | 138 | 110 | 1.35 |
| 28 | 7.45 | 32 | 109 | 22 | -1.2 | 0.9 | 4.3 | 4.1 | 136 | 109 | 1.37 |
| 35 | 7.45 | 28 | 65 | 21 | -4 | 1 | 4.8 | 4.2 | 136 | 109 | 1.71 |
| 41 | 7.39 | 36 | 55 | 21.8 | -2.5 | 1 | 4.2 | 4 | 137 | 109 | 1.58 |
| 50 | 7.5 | 29 | 86 | 20.1 | 0.1 | 0.8 | 4 | 3.9 | 139 | 108 | 1.35 |
| 61 | 7.35 | 36 | 89 | 21.8 | -5.1 | 0.7 | 5.2 | 4.5 | 136 | 109 | 1.34 |
| 68 | 7.39 | 37 | 70 | 21.8 | -2.3 | 0.7 | 4.5 | 4.7 | 136 | 109 | 1.3 |
| 109 | 7.38 | 38 | 96 | 22.5 | -2 | 1 | 4.8 | 4.4 | 135 | 109 | 1.33 |
| 118 | 7.46 | 29 | 100 | 21 | -2.5 | 2.3 | 5.4 | 4.5 | 138 | 110 | 1.28 |
| 120 | 7.4 | 32 | 98 | 22 | -4 | 0.9 | 5.1 | 4.4 | 135 | 109 | 1.33 |
| 133 | 7.47 | 30 | 94 | 20.8 | -0.8 | 1.6 | 7 | 4.2 | 135 | 109 | 1.24 |

PCO_2_: partial pressure of carbon dioxide, PO_2_: partial pressure of oxygen, HCO3^-^: bicarbonate
