# Supplementary material for: Case report of successful conservative management of neonatal gastrointestinal perforation and intestinal stenosis
Source: Medicine (Baltimore). 2026 Sep 18;105(38):e50502. doi: 10.1097/MD.0000000000050502 (PMC13592972; doi:10.1097/MD.0000000000050502)
Supplement: Supplementary file 4 [file medi-105-e50502-s004.docx]

| Supplementary table 4 Biochemistry results during the patient's hospitalization | | | | | | | | |
| --- | --- | --- | --- | --- | --- | --- | --- | --- |
| Days after admission | ALT (U/L) | AST (U/L) | TP (g/L) | ALB (g/L) | TBIL (μmol/L) | DBIL (μmol/L) | IBIL (μmol/L) | CREA (μmol/L) |
| 1 | 6 | 22 | 33.6 | 21.8 | 102.7 | 14.2 | 88.5 | 36 |
| 3 | 56 | 36 | 45.0 | 29.8 | 98.4 | 10.5 | 87.9 | 21 |
| 9 | 13 | 31 | 43.7 | 28.2 | 40.0 | 19.6 | 20.4 | 15 |
| 32 | 11 | 24 | 47.8 | - | 19.0 | - | - | 19 |
| 54 | 23 | 23 | 50.5 | - | 28.7 | - | - | 15 |
| 80 | 53 | 56 | 56.1 | 43.7 | - | - | - | 25 |
| 109 | 74 | 51 | 42.6 | - | 4.5 | - | - | 17 |
| 136 | 29 | 40 | 43.4 | 35.1 | 2.3 | 0.5 | 1.8 | 19 |

ALT: alanine aminotransferase, AST: aspartate aminotransferase, TP:	total protein, ALB: albumin, TBIL:	total bilirubin, DBIL:	direct bilirubin, IBIL:	indirect bilirubin, CREA:	creatinine
